# Fetal respiratory movements improve reliability of heart rate variability and suggest a coupling between fetal respiratory arrhythmia and vagal activity

**DOI:** 10.14814/phy2.15224

**Published:** 2022-03-21

**Authors:** Anne Rahbek Zizzo, Ida Kirkegaard, Camille From Reese, John Hansen, Niels Uldbjerg, Henning Mølgaard

**Affiliations:** ^1^ Department of Obstetrics and Gynaecology Aarhus University Hospital Aarhus N Denmark; ^2^ Department of Health Science and Technology Aalborg University Aalborg Denmark; ^3^ Department of Cardiology Aarhus University Hospital Aarhus N Denmark

**Keywords:** autonomic nervous system, fetal heart rate variability, fetus, gestational age, reliability, respiratory sinus arrhythmia, spectral domain, time domain, vagal activity

## Abstract

Fetal heart rate variability (FHRV) reflects autonomic cardiac regulation. The autonomic nervous system constantly adjusts the heart rate to maintain homeostasis. By providing insight into the fetal autonomic state, FHRV has the potential to become an investigational and clinical instrument. However, the method needs standardization and the influence of fetal movements, including fetal respiratory movements, is not well explored. Therefore, in a highly standardized setting, the aim was to evaluate the association between fetal movements and fetal heart rate variability (FHRV) including their impact on reliability. Fetal heart rate was obtained by noninvasive fetal electrocardiography (NI‐FECG) and fetal movements by simultaneous ultrasound scanning in 30 healthy singleton pregnant women on two occasions with a maximum interval of 7 days. The standard deviation of normal‐to‐normal RR‐intervals (SDNN), root mean square of successive RR‐interval differences (RMDDS), high‐frequency power (HF‐power), low‐frequency power (LF‐power), and LF/HF were measured. A multivariate mixed model was used and reliability was defined as acceptable by a coefficient of variance (CV) ≤15% and an intraclass correlation coefficient (ICC) ≥0.80. During time periods with fetal respiratory movements, the highest reliability was achieved. Intra‐ and inter‐observer reliability measurements were very high (CV: 0–9%; ICC ≧ 0.86). Within the same recording, SDNN and RMSSD achieved acceptable reliability (CV: 14–15%; ICC ≧ 0.80). However, day‐to‐day reliability displayed high CV’s. In time periods with fetal respiratory movements, as compared to periods with quiescence RMSSD and HF‐power were higher (Ratio: 1.33–2.03) and LF/HF power lower (Ratio: 0.54). In periods with fetal body movements SDNN, RMSSD and HF‐power were higher (Ratio: 1.27–1.65). In conclusion, time periods with fetal respiratory movements were associated with high reliability of FHRV analyses and the highest values of parameters supposed to represent vagal activity.

## Graphical abstract

Fetal heart rate variability obtained by non‐invasive fetal electrocardiography needs standardization. Adding fetal movements assessed by ultrasound, revealed high reliability and a respiratory dependent sinus arrhythmia.

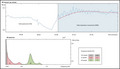

## 1 INTRODUCTION

Fetal heart rate variability (FHRV) based on high accuracy beat‐to‐beat detection, reflects the fetal autonomic cardiac regulation and, indirectly, the autonomous response. This may be valuable in the understanding of fetal neurophysiology, as well as in the aspect of surveillance of compromised fetuses, where the existing surveillance, primarily based on Doppler ultrasound flows, mainly reflects the fetal cardiovascular adaptation to intrauterine hypoxia.

In adults, the well‐known vagally mediated respiratory sinus arrhythmia (RSA) is a strong positive predictor of health (Rajendra Acharya et al., 2006; Task Force, 1996). Time domain and spectral domain parameters display RSA and other sinus rhythms of heart rate (Eckberg, 1983; Hayano et al., 1991). In order to standardize analyses, rest and paced respiration are prescribed during the evaluation of adults, as HRV is strongly impacted by physical movements. Current evidence in fetuses also suggests an association between FHRV and fetal movements; however, studies in FHRV are heterogeneous and the method for FHRV needs standardization and validation (ref review).

Fetal movements are unpredictable and constantly changing, which is why the strict criteria, recommended in adults, are impossible to directly transfer to fetuses. To overcome this challenge, heart rate pattern (HRP), which is based on fetal behavioral states (FBS) as defined by Nijhuis et al. (1982), has been applied in many studies (van Laar et al., 2009; Schneider et al., 2008, 2009). FBS and HRP reflect to some extent fetal movements and fetal state (Nijhuis et al., 1982; Pillai & James, 1990a; Pillai et al., 1992). However, fetal movements and especially respiratory movements may occur in all HRP (Pillai & James, 1990b). Ultrasound on the other hand, provides the opportunity for a continuous evaluation of fetal movements (Marsal, 1983).

By this method, Arias‐Ortega et al. found higher values of parameters reflecting the RSA in the small for gestational age (SGA) fetus compared to the average for gestational age (AGA) fetus. However, this was only the case during fetal respiratory movements (FRM) (Arias‐Ortega et al., 2016). A more detailed understanding of the relation between FHRV and fetal movements may therefore also carry important information in regard of the pathophysiology of fetal compromise.

Factors other than fetal movements may also affect FHRV, including gestational age (Van Leeuwen et al., 2003; Schneider et al., 2018), maternal position (Stone et al., 2017), maternal smoking (Kapaya et al., 2015; Spyridou et al., 2017) maternal caffeine intake (Koenig et al., 2013), maternal exercise (Ref van leuween), maternal ethnicity (Tagliaferri et al., 2017), and fetal sex (Bernardes et al., 2008; Goncalves et al., 2017). These factors also need consideration when standardizing the method for FHRV.

Therefore, standardizing the method used in FHRV assessment is essential as high reliability is crucial from a clinical perspective. From a physiological perspective, absolute reliability is interesting, as it adds information on the variance within each fetus and thereby the complexity of the regulation of the fetal heart rate within the fetus, while the relative reliability adds information on the variance between fetuses in relation to the variance within fetuses.

The influence of fetal movements on FHRV, including their impact on reliability, therefore needs evaluation in a standardized method. The aim of this study was to evaluate the association between fetal movements and FHRV including their impact on reliability in healthy fetuses.

## 2 METHODS

### 2.1 Ethical approval

The study conformed to the standards set by the *Declaration of Helsinki* and have been approved by the Danish Data Protection Agency (1–16–02–440–15) and the Danish National Committee on Health Research Ethics (1–10–72–227–15). Written and informed consent was obtained.

### 2.2 Participants

In this observational cohort study, we included healthy singleton pregnant women at the Region Hospital of Horsens, Denmark.

All participating women attained the prenatal screening program consisting of two ultrasound scans, providing determination of estimated due date and screening for fetal chromosomal anomalies and malformations. Exclusion criteria were obstetric complications before or at inclusion, chromosomal anomalies, fetal malformations, and growth restriction diagnosed in utero.

Thirty women divided into three gestational age (GA) groups were included in the analyses: group A) 20^+0^ to 27^+6^ (A_20‐27_); group B) 28^+0^ to 34^+6^ (B_28‐34_); group C) 35^+0^ to 41^+0^ (C_35‐41_). Only women displaying visible fetal R‐waves in two noninvasive fetal electrocardiography (NI‐FECG) recordings were eligible for inclusion in the analyses. The NI‐FECG was obtained on two occasions with a maximum interval of 7 days.

Maternal caffeine intake, smoking, and high‐intensity exercise were registered as hours since the last exposure. Women confirming one of these exposures on day 1 were requested to obtain the same exposure on day 2.

The NI‐FECG recordings from day 1 have been included in a former study of reliability and heart rate pattern (HRP) (Zizzo et al., 2020). However, in that study, fetal movement detection by ultrasound was not considered.

### 2.3 Acquisition of NI‐FECG

**FIGURE 1:**
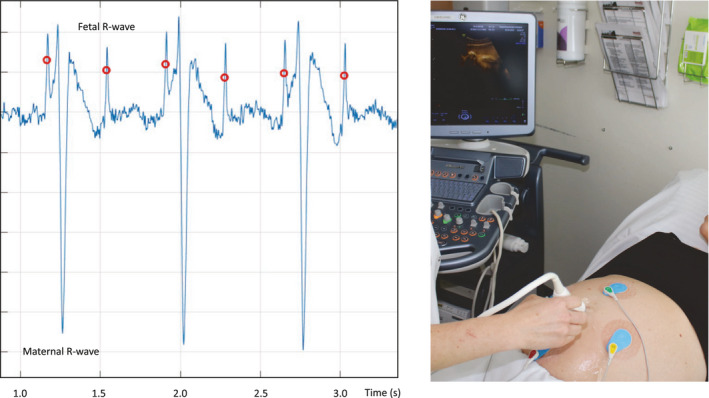
An example of fetal electrocardiography, obtained by electrodes placed on the maternal abdomen, and simultaneous detection of fetal movements, obtained by ultrasound. The automatic fetal R‐wave detection is marked by red dots. (with permission from the depicted persons)

During the acquisition of NI‐FECG, the pregnant woman was placed in a supine or lateral resting position in a quiet room. A 20‐minute NI‐FECG was obtained by four electrodes (Ag/AgCl) and one ground electrode placed on the maternal abdomen. All acquisitions were performed by the same NI‐FECG device (Viewcare A/S, Søborg, Denmark) during the daytime (8 a.m.–4 p.m.) with a resolution of 24‐bit and a sampling frequency of 1 kHz. A 50 Hz notch, together with 5 Hz low‐ and 150 Hz high‐pass filters were also applied. The algorithm for automatic fetal R‐wave detection was based on templates of fetal and maternal QRS‐complexes (Viewcare A/S, Søborg, Denmark) (Sæderup, 2019) (Figure 1).

Fetal B‐mode ultrasound scanning (Voluson E10 and Voluson S6, GE Healthcare) was performed continuously with the NI‐FECG. Fetal movement pattern was divided into three categories: (1) fetal body movements (FBM): covering period of continuous FBM (extremities and/or trunk); (2) FRM: covering continuous movements of the thoracic diaphragm, without any movements of the fetal extremities and trunk; (3) fetal quiescence (FQ): no fetal body or respiratory movements were allowed, except occasional kicks or startles. Every 15‐second epoch of the NI‐FECG recording was classified into one of these three categories of movement patterns. Ultrasound scans and classification of fetal movement patterns were performed by the same observer throughout the study.

### 2.4 Processing

Kubios Premium (Kubios heart rate variability software version 3.3; Biosignal Analysis and Medical Imaging Group, Department of Physics, University of Kuopio, Kuopio, Finland) was used for the FHRV analyses. Detrending based on smoothen priors regulation was performed (Tarvainen et al., 2002) with the smoothing parameter set to 500, corresponding to a cut‐off frequency at 0.035 Hz (Tarvainen et al., 2014). Artifact correction relied on the Cubic Spline interpolation (Daskalov & Christov, 1997; Mateo & Laguna, 2000), and a threshold of 40 ms was appropriate in most recordings. RR‐intervals deviating more than 40 ms from the preceding RR‐interval were thereby removed and replaced. However, in a few recordings, a threshold of 100 ms was needed. Additionally, time series were systematically and manually checked for errors in the artifact correction. A correction of maximum 5% of each time series was allowed. In the spectral analyses, RR intervals were re‐sampled at 4 Hz.

### 2.5 Selection of time series

**FIGURE 2:**
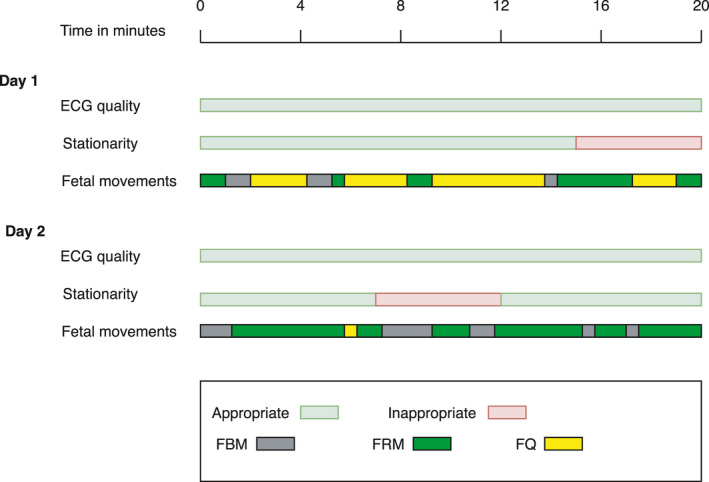
Inclusion criteria for selection of time series, depicted by an example showing the quality of fetal electrocardiography (ECG quality), stationarity of mean RR (SHRP) (Stationarity), and the categorization of fetal movements (Fetal movements). In this case, the prevailing fetal movement pattern deviates from day 1 to day 2. Appropriate ECG quality: maximum 5% correction of non‐normal fetal RR‐intervals Appropriate stationarity: No accelerations or decelerations (± 15 beats per minute (bpm) /15 seconds) and floating of baseline less than 10 bpm per 2 minutes (stationary heart rate pattern (SHRP)). Fetal movements obtained by ultrasound: FBM: Fetal body movements (continuous FBM of extremities, trunk). FRM: Fetal respiratory movements (continuous movements of the thoracic diaphragm, without any other movements). FQ: Fetal quiescence (no fetal body or respiratory movements, except occasional and feeble sparkles)

Time series included in the analyses fulfilled three predefined criteria: (1) Maximum 5% correction of RR‐intervals due to artifacts, missing beats or extrasystoles; (2) Stationarity of mean RR‐intervals (mean RR), which was evaluated from a cardiotocography‐like pattern (Tachogram) and defined as no accelerations or decelerations (± 15 beats per minute (bpm)/15 s) and floating of baseline less than 10 bpm per 2 min (stationary heart rate pattern [SHRP]) (Zizzo et al., 2020; (3) One of the three categories of fetal movement pattern (FBM, FRM, or FQ). However, in the analyses where fetal movements were ignored, time‐series only fulfilled criteria (1) and (2) (Figure 2).

Short time series of 64 seconds was chosen to increase the chance of obtaining time series containing only one of the categories of fetal movement patterns. These short times series have been used previously (van Laar et al., 2010, 2011, 2014) and have been documented to be sufficient for reliable FHRV analysis (Zizzo et al., 2020).

### 2.6 Reliability measurements

Selection of time series was performed by three observers. Intra‐ and inter‐observer reliability measurements were based on 10 of the recordings containing at least four minutes of FRM and another 10 recordings containing at least four minutes of FQ. The three observers independently selected all appropriate time series according to the criteria described above and repeated this selection in the same recordings at least 6 weeks later. All other selections of time series were performed by the same observer throughout the study. The inter‐observer reliability measurement was based on the three observers on their first day of selection, while the intra‐observer reliability measurement was based on each combination of observer and recording (fetus) on their first and second day of selection.

The same‐recording reliability was based on all recordings containing at least four‐time series from one specific fetal movement pattern. The mean of the first half (at least two) of the time series was compared to the mean of the last half of the time series, in the same recording.

The day‐to‐day reliability was based on recordings from two different days, in the same fetus. Only fetuses displaying at least two‐time series from one specific fetal movement pattern at both day 1 and day 2 were included in these analyses. The mean values from day 1 were compared to the mean values from day 2.

### 2.7 Analyses of fetal heart rate variability

Time domain analyses included: mean RR‐interval (mean RR [ms]), a standard deviation of normal to normal RR‐intervals (SDNN [ms]), and the root mean square of successive RR‐interval differences (RMSSD (ms). Spectral analyses included: high‐frequency power (HF‐power [ms^2^]), low‐frequency power (LF‐power [ms^2^]), and LF‐power/HF‐power). Both fast Fourier transformation (FFT) and the autoregressive model (AR) with the order set to 24, were used (Task Force, 1996).

The frequency bands were set to: LF‐power (0.04–0.4 Hz), HF‐power (0.4–1.5 Hz) (Groome et al., 1994; Gustafson et al., 2011), based on the frequency of FRM (Dornan et al., 1984), as well as former studies indicating an HF‐peak around 0.7 (Divon et al., 1985; Zizzo et al., 2020).

RMSSD and HF power are generally interpreted as vagally mediated parameters, whereas SDNN and LF power probably reflect both sympathetic and parasympathetic activity (Task Force, 1996); however, these assumptions are elaborated further in the discussion.

### 2.8 Statistics

All variables were normally distributed and homoscedastic on the logarithmic scale.

The multivariate mixed model was used due to repeated measurements within the same fetus (day 1 and day 2) and within the same recording (same analyses). Fetus and the combination of fetus and recording day as the random effect was used to adjust for correlation within fetuses and recording days.

The median with 95% CI, coefficient of variation (CV), intraclass correlation coefficient (ICC) and 95% limits of agreement (LoA) were estimated assuming the same standard deviation (SD) in the groups compared. ICC, CV, and LoA were performed to estimate (a) intra‐observer reliability; (b) inter‐observer reliability; (c) the same recording reliability; and (d) day‐to‐day reliability.

CV is defined as follows:

$$CV=\surd (\exp\left(\sigma^{2}\right)‐1)$$

ICC is defined as follows:

$$ICC=\sigma_{B}^{2}\;/\left(\sigma_{B}^{2}+\sigma_{E}^{2}\right)$$

where $\sigma_{B}^{2}$ denotes the between subject variance and $\sigma_{E}^{2}$ the within random error.

ICC (relative reliability) was defined as poor (ICC <0.4), moderate (0.4 ≤ ICC <0.6), good (0.6 ≤ ICC <0.8), and excellent (ICC ≥0.8). Based on former studies acceptable reliability was interpreted as a CV ≤15% and an ICC ≥0.8 (Atkinson & Nevill, 1998; Pinna et al., 2007; Sillesen et al., 2019; Sookan & McKune, 2012).

LoA is defined as follows:

LoAratio=exp(μ±1.96∙σ)

The multivariate mixed model was also used to estimate the difference between FRM and FQ. The development through gestational age was added into the model as a fixed effect. Due to few measurements containing FBM, the comparison to FQ in the second trimester was performed without taking gestational age into the model. The medians with 95% CI and ratios with 95% CI are given.

**FIGURE 3:**
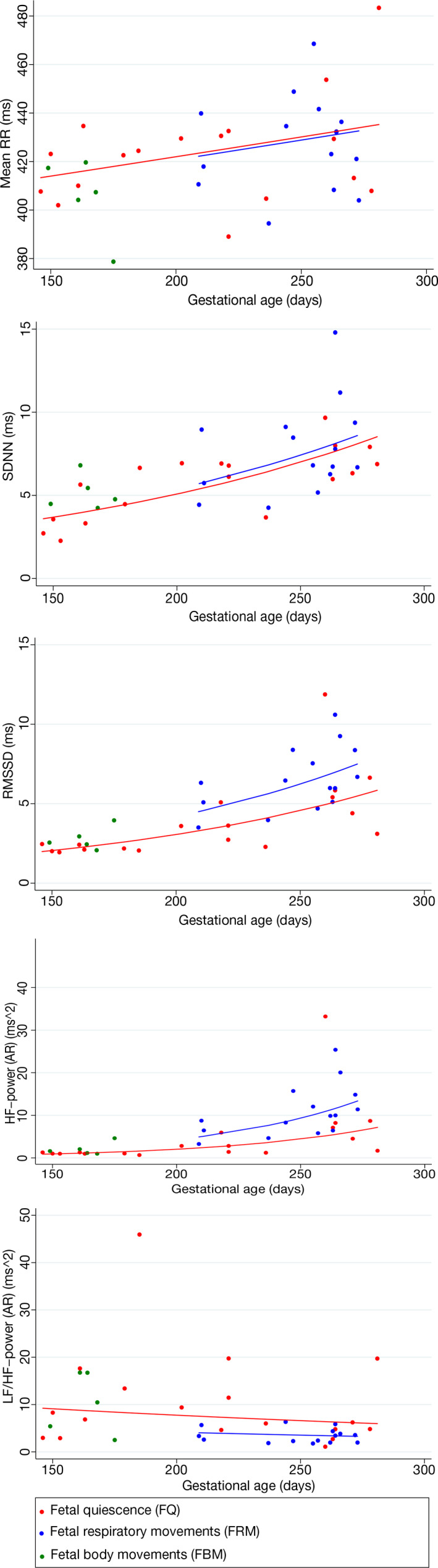
Scatterplots and median curves for mean RR, SDNN, RMSSD, HF‐power (AR), and LF/HF‐power (AR) in relation to gestational age and fetal movements. Median curves for the outcomes Mean RR, SDNN, RMSSD, HF‐power (AR), and LF/HF‐power (AR) as a function of independent variables, gestational age, and fetal movements were obtained using a linear mixed model for the logarithm of the outcome data and back‐ transforming the result on the original outcome scale using the exponential function. The assumption of linearity of log‐outcome data as a function of the independent variables was validated using residual plots. Due to few observations, no median curve is shown for fetal body movements (FBM)

All data are shown in scatterplots (Figure 3) Research data are not shared due to privacy.

## 3 RESULTS

A total of 44 women were included in the study (Table 1): 10 in the GA group A_20–27_, 18 in the GA group B_28–34_, and 16 in the GA group C_35–41_. Among these, 30 women (10 in each GA group) displayed visible fetal R‐waves in both day 1 and day 2 recordings and were therefore included in the analyses. However, 10 of the 14 excluded women actually displayed visible fetal R‐waves in one of the two recordings.

**TABLE 1:** Characteristics of included women

	All included women *n* (44)	Women included in analyses^a^ *n* (30)
Maternal age (years) Mean (95%CI)	29.4 (27.8; 31.0)	28.7 (27.2; 30.3)
Maternal BMI (kg/m^2^) Mean (95%CI)	24.3 (22.1; 25.7)	23.8 (22.2; 25.3)
Nulliparity *n* (%)	28 (63.6)	20 (66.7)
Daily caffeine consumption *n* (%)	7 (15.9)	5 (16.7)
Daily high‐intensity exercise *n* (%)	2 (4.5)	2 (6.7)
Smoking *n* (%)	2 (4.5)	2 (6.7)
Fetal gender (male) *n* (%)	28 (63.6)	16 (53.3)
Birthweight below −2SD *n* (%)	1 (2.3)	1 (3.3)
GA (group I) Median (range)	23.1 (20,9; 25.6)	23.1 (20.9; 25.6)
GA (group II) Median (range)	30.7 (28.3; 34.6)	31.4 (28.9; 34.6)
GA (group III) Median (range)	37.6 (35.3; 40.0)	37.7 (36.1; 40.0)

Abbreviations: BMI, body mass index; GA, gestational ageSD, standard deviation.

^a^ Only women with high quality NI‐fECG were included in the analyses.

One neonate from the GA group C_35–41_ demonstrated a birth weight <2.3 percentile of mean weight for gestational age; however, the neonate did not need admission or showed any signs of dysmaturity. No severe birth complications were reported, but two neonates were admitted to the neonatal intensive care unit (NICU); one due to postpartum sepsis and one due to transient signs of birth asphyxia. These neonates were kept in the analyses as complications developed during delivery or after, and therefore are unlikely to have had any influence at the time of recording.

No malformations were diagnosed post‐partum.

In GA group A_20–28_ (2nd trimester), only time series categorized as FQ and FBM was found. Periods of FRM were very short and rare in this early GA group and therefore not included in the analyses. In GA B_28–34_ and C_35–41_ (3rd trimester) analyses were restricted to time series categorized as FQ and FRM, as time series fulfilling the criteria of stationarity were missing during FBM in the third trimester.

In the third trimester (GA group B_28–34_ and C_35–41_), 62% of all recordings contained at least two appropriate time series of FRM, while this number was 56% for FQ. In the second trimester (GA group A_20–27_), 84% of recordings contained at least two appropriate time series of FQ and 65% of FBM. Of the total recording time, 35% was included in analyses of either: FRM (19%) or FQ (16%) in the 3rd trimester, or FBM (15%) and FQ (20%) in the 2nd trimester.

In the analyses, which only fulfilled SHRP and less than 5% correction of artifacts and not were restricted to fetal movement pattern, 97% of recordings contained at least two appropriate time series and 48% of all recording time was included in these analyses.

### 3.1 Reliability of FHRV in relation to fetal movements

Intra‐observer reliability measurements were very high in both time domain and AR spectral parameters. Thus, the CVs were well below 15% and between 0–7% during FRM. All intra‐observer ICC were above 0.98. (Table 2). Inter‐observer reliability disposed CVs ≤15% and between 0 and 9% during FRM. All inter‐observer ICC were ≥0.96 for both time domain and AR spectral parameters. FFT spectral parameters performed less positive showing lower intra‐ and inter‐observer reliability compared to time domain and AR spectral parameters (during FRM; intra‐observer CV: 6–12%; inter‐observer CV: 5–15%; all ICC ≥0.86).

**TABLE 2:** Reliability of time domain and spectral domain (Auto Regressiv model) parameters in the 3rd trimester (GA 28^+0^– 41^+0^), divided into fetal movement pattern

Fetal respiratory movements (FRM)												
	CV, same recording (*n* = 15)	CV, day‐to‐day (*n* = 7)	CV, day‐to‐day^a^ (*n* = 5)	CV, intra‐ observer (*n* = 10)	CV, inter‐observer. (*n* = 10)	95% LoA, same day	95% LoA, day‐to‐day	ICC, same day	ICC, day‐to‐day	ICC, day‐to‐day^a^	ICC, intra‐observer	ICC, inter‐observer
	%	%	%	%	%	Ratio	Ratio					
Mean RR	**2 (1;3)**	**3 (2;5)**	**3 (2;4)**	**0 (0; 0)**	**0 (0;0)**	0.97;1.06	0.90;1.08	**0.85**	0.44	error	**1.00**	**1.00**
SDNN	**14 (10;20)**	24 (14;41)	21 (11;41)	**3 (0; 6)**	**3 (2; 4)**	0.77;1.55	0.59;0.90	**0.85**	0.76	0.77	**0.99**	**0.99**
RMSSD	**15 (10;21)**	27 (16;48)	**14 (8;27)**	**4 (2; 6)**	**3 (2; 4)**	0.67;1.54	0.43;1.36	**0.80**	0.69	**0.83**	**0.99**	**0.99**
LF‐power (AR)	34 (24;50)	61 (34;121)	63 (32;146)	**4 (0; 9)**	**6 (6; 11)**	0.55;2.90	0.22;1.10	**0.83**	0.71	0.68	**1.00**	**1.00**
HF‐power (AR)	29 (20;46)	64 (36;129)	31 (17;62)	**5 (0; 12)**	**8 (6; 11)**	0.46;2.34	0.16;2.08	0.76	0.61	0.78	**1.00**	**0.99**
LF/HF‐power (AR)	36 (25;53)	68 (44;113)	67 (34;160)	**7 (2; 16)**	**9 (7; 13)**	0.49;3.03	0.14;5.53	0.52	0.00	0.09	**1.00**	**0.96**

Fetal quiescence (FQ)												
	CV, same ecording (*n *= 11)	CV, day‐to‐day (*n* = 11)	CV, day‐to‐day^a^ (*n* = 10)	CV, intra‐observer^b^ (*n* = 10)	CV, inter‐observer^b^ (*n* = 10)	95% LoA, same day	95% LoA, day‐to‐day	ICC, same day	ICC, day‐to‐day	ICC, day‐to‐day^a^	ICC^b^, intra‐observer	ICC^b^, inter‐observer
	%	%	%	%	%	Ratio	Ratio					
Mean RR	**2 (1;3)**	**5 (3;8)**	**1 (1;2)**	**0 (0;0)**	**0 (0;0)**	0.94;1.06	0.85;1.11	**0.88**	0.44	**0.94**	**1.00**	**0.00**
SDNN	23 (15;35)	31 (20;48)	21 (13;33)	**4 (1;8)**	**5 (3;6)**	0.54;1.96	0.38;1.84	0.38	0.77	**0.88**	**0.98**	**0.98**
RMSSD	18 (12;27)	25 (16;38)	16 (10;25)	**4 (2; 6)**	**3 (2;4)**	0.59;1.63	0.46;1.83	**0.87**	**0.85**	**0.93**	**0.99**	**0.99**
LF‐power (AR)	54 (34;89)	69 (43;120)	41(26;67)	**9 (0;19)**	**11 (8;15)**	0.26;4.73	0.13;3.54	0.27	0.79	**0.91**	**0.97**	**0.98**
HF‐power (AR)	32 (21;51)	42 (27;68)	30 (19;48)	**6 (0;13)**	**13 (10;18)**	0.38;2.35	0.28;2.76	**0.90**	**0.88**	**0.93**	**0.99**	**0.98**
LF/HF‐power (AR)	41 (27;66)	42(27;67)	37 (24;60)	**9 (2;20)**	**15 (11;21)**	0.39;3.56	0.27;2.23	**0.80**	**0.85**	**0.89**	**0.97**	**0.97**

Fetal movements not registered												
	CV, same recording. (*n* = 37)	CV, day‐to‐day (*n* = 25)	CV, day‐to‐day^a^ (*n* = 22)	CV, intra‐observer^b^ (*n* = 20)	CV, inter‐observer^b^ (*n* = 20)	95% LoA, same day	95% LoA, day‐to‐day	ICC, same day	ICC, day‐to‐day	ICC, day‐to‐day^a^	ICC, intra‐observer^b^	ICC, inter‐observer^b^
	%	%	%	%	%	Ratio	Ratio					
Mean RR	**2 (2;2)**	**4 (3;6)**	**4 (3;5)**	**0 (0; 0)**	17 (9;22)	0.96;1.06	0.88;1.08	**0.99**	0.49	0.63	**1.00**	**1.00**
SDNN	19 (15;24)	30(22;40)	25 (19;35)	**4 (0; 8)**	**3 (2;4)**	0.68;1.79	0.40;1.96	**0.83**	0.64	0.71	**0.98**	**0.98**
RMSSD	17 (13;21)	38(28;51)	33 (24;45)	**4 (3; 8)**	**3 (2;4)**	0.65;1.63	0.34;2.71	**0.86**	0.54	**0.83**	**0.99**	**0.99**
LF‐power (AR)	45 (35;58)	65 (47;92)	55 (40;78)	**7 (1;14)**	**11 (9;14)**	0.42;3.84	0.16;3.65	**0.81**	0.69	0.74	**0.99**	**0.98**
HF‐power (AR)	36 (28;46)	85 (61;126)	74 (52;109)	**6 (0; 13)**	**9 (8;12)**	0.39;2.76	0.12;8.01	**0.83**	0.53	0.61	**0.99**	**0.99**
LF/HF‐power (AR)	42 (33;54)	56 (41;78)	56 (40;79)	**8 (5;15)**	**11 (9;14)**	0.43;3.51	0.19;3.01	0.74	0.62	0.65	**0.99**	**0.97**

Note

Mean RR, SDNN, RMSSD provided in ms. LF‐power, HF‐power provided in ms^2^.

Values defined as acceptable are highlighted by bold.

Abbreviations: CV, coefficient of variance; ICC, intraclass correlation coefficient; LoA, limits of agreement.

^a^ Based on fetuses demonstrating the same prevailing fetal movement pattern at Day 1 and Day 2.

^b^ GA 20^+0^– 40^+0^.

In the third trimester, the reliability within the same recording was high in time domain parameters (mean RR, SDNN, RMSSD), particularly in time series containing FRM (Table 2) displaying CVs ≤15% and ICC’s ≥ 0.80. In time series containing FQ, RMSSD reached a CV at 18% and ICC at 0.87, while SDNN performed less favorable. Spectral parameters, on the other hand, displayed low absolute reliability in all movement patterns indicated by high CVs around 30% in the best performing parameters related to FRM. Nevertheless, ICCs were mostly “good” (0.6 ≤ ICC <0.8) and “excellent” (ICC ≥0.80) in spectral analyses. In the second trimester (GA group A_20–27_), the same‐recording reliability during FBM demonstrated high CVs, but also some high ICCs in the categories “good” (0.6 ≤ ICC <0.8) and “excellent” (ICC ≥0.80) (Table 3). However, these findings are based on few observations.

**TABLE 3:** Reliability of time domain and spectral domain (Auto Regressiv model) parameters in the 2nd trimester (GA 20^+0^–27^+6^), divided into fetal movement pattern

	Fetal body movements (FBM)		Fetal quiescence (FQ)		Unrestricted fetal movements	
	CV same recording *n* (5)	ICC same recording	CV same recording (*n* = 7)	ICC same recording	CV same recording (*n* = 15)	ICC same recording
	%		%		%	
Mean RR (ms)	1 (0;2)	0.96	1 (1;2)	0.83	1 (1;2)	0.92
SDNN (ms)	23 (12.44)	0.16	19 (11;33)	0.78	20 (14;28)	0.84
RMSSD (ms)	17 (9;31)	0.62	23 (15–34)	0.00	24 (17;35)	0.71
LF‐power (ms^2^)	53 (27;116)	0.44	45 (26;82)	0.78	39 (27;58)	0.87
HF‐power (ms^2^)	30 (16;60)	0.79	54 (36;87)	0.00	52 (35;79)	0.64
LF/HF‐power (ms^2^)	41 (21;84)	0.75	86 (46;197)	0.55	45 (31;68)	0.75

Abbreviations: CV, coefficient of variance; ICC, intraclasscorrelation coefficient.

Day‐to‐day reliability was lower than the same‐recording reliability for all parameters. Still, SDNN and RMSSD were the best performing parameters (CVs at 24–31% and ICC at 0.69–0.85). By restricting the day‐to‐day analyses to fetuses demonstrating the same prevailing fetal movement pattern on day 1 and day 2, reliability increased in most parameters and became comparable with the same‐recording analyses (Table 2 and Figure 2).

### 3.2 Association between fetal movements and FHRV

For all three categories of the fetal movement patterns, the magnitude of all FHRV parameters increased through gestational age except from LF/HF‐power which decreased (Figure 3). On a log‐scale, these associations were linear and displayed the same slopes.

In the second trimester, FBM was associated with an increase in SDNN, RMSSD, and HF‐power (ratios: 1.27–1.65) and a decrease in Mean RR (Ratio 0.98), whereas LF/HF‐power was unaffected (ratio 0.84; *p* = 0.66) when compared to FQ (Figure 3 and Table 4).

**TABLE 4:** Time domain and spectral domain (Auto Regressive model) parameters divided into fetal movements in relation to gestational age

Parameters	Gestational age: week 20–27				Gestational age: week 28–41			
	FBM	FQ	FBM/FQ		FRM	FQ	FRM/FQ	
	Median (SD) (95% CI)^a^	Median (SD) (95% CI)^a^	Ratio (95% CI)^b^	*p*‐value	Median (SD) (95% CI)^a^	Median (SD) (95% CI)^a^	Ratio (95% CI)^b^	*p*‐value
Mean RR (ms)	408 (1.0) (398;420)	414 (1.0) (404;426)	0.98 (0.97; 1.00)	**0.01**	430 (1.0) (418;442)	429 (1.0) (417;441)	1.00 (0.97;1.26)	0.83
SDNN (ms)	4.9 (1.3) (3.8;6.2)	3.9 (1.3) (3.0;4.9)	1.27 (0.97; 1.65)	0.08	7.9 (1.2) (6.6;9.5)	6.9 (1.2) (5.8;8.2)	1.06 (0.86; 1.31)	0.58
RMSSD (ms)	2.7 (1.2) (2.3;3.2)	2.2 (1.2) (1.9;2.5)	1.25 (1.06; 1.48)	**0.01**	5.9 (1.3) (4.7;7.4)	4.6 (1.3) (3.7;5.7)	1.33 (1.04; 1.71)	**0.03**
LF‐power (ms^2^)	13.1 (1.9) (7.5;23.0)	9.6 (1.9) (5.5;16.7)	1.37 (0.78; 2.40)	0.28	30.5 (1.6) (20.7;45.1)	29.5 (1.6) (20.0;43.5)	1.00 (0.65; 1.53)	0.98
HF‐power (ms^2^)	1.70 (1.5) (1.3;2.5)	1.0 (1.5) (0.7;1.5)	1.65 (1.13; 2.42)	**0.01**	6.4 (1.6) (4.0;10.3)	4.5 (1.6) (2.9;7.0)	2.03 (1.22; 3.37)	**0.01**
LF/HF‐power (ms^2^)	7.8 (2.1) (3.8;16.0)	9.3 (2.1) (4.6;18.8)	0.84 (0.38; 1.84)	0.66	3.5 (1.7) (2.5;5.0)	6.5 (1.7) (4.5;9.4)	0.54 (0.37; 0.79)	**0.00**

Significant *p*‐values are highlighted by bold.

Abbreviations: 95% CI, 95% confidence interval; FBM, fetal body movements; FQ, fetal quiescence; FRM, fetal respiratory movements; SD, standard deviation.

^a^ Medians develop through gestational age and medians as a function of gestational age are shown in Figure 3.

^b^ Ratios in gestational age: week 28–41: The development in GA through the 3rd trimester is included in the mixed model as a fixed effect.

In the third trimester, FRM was associated with an increase in RMSSD and HF‐power (ratios: 1.33–2.0) and a decrease in LF/HF‐power (ratio: 0.54) when compared to FQ (Figure 3 and Table 4).

## 4 DISCUSSION

### 4.1 Main results

The use of FHRV as an investigational tool and clinical measure requires standardization. We find that by careful selection of time series used for analysis, related to fetal movements, it is possible to obtain laboratory‐like conditions with acceptable reliability, especially during FRM.

When comparing time series from FRM with time series from FQ, we found increased RMSSD, increased HF power, and decreased LF/HF power.

### 4.2 Strengths and limitations

We sought to attain close to “laboratory” conditions by standardizing the setting using the same, NI‐FECG device, ultrasound device, and recording time (daytime) in all recordings. Furthermore, we standardized maternal position during recordings, maternal caffeine consumption, maternal exercise, and hours since smoking. These standardizations, combined with the accurate detection of R‐waves (1 kHz sampling frequency), systematic correction of artifacts, allowing a maximum of 5% corrections, and continuous detection of fetal movements, bring consistency into our results. However, by using these strict inclusion criteria, the rate of recordings excluded in each category of fetal movement pattern spanned from 16% to 44% depending on gestational age and movement pattern, but longer recordings may solve this challenge.

Fetal behavioral state cannot explain our findings. Moreover, adjusting for behavioral state or HRP may increase the significance of our findings. Fetal behavioral state, which is closely linked to HRP may be an important source of confounding. Therefore, all included time series were classified into HRP (Schneider et al., 2008) and a tendency towards a higher rate of time series from HRP I was seen in the analyses of FRM as compared to FQ. HRP I is associated to lower RMSSD, SDNN, and HF power (Frank et al., 2006; van Laar et al., 2014; Stone et al., 2017) and we find an increase in RMSSD and HF power during FRM.

We do not consider fetal sex as a confounder, as former studies found no significant difference in fetal behavior between male and female fetuses (Robles de Medina et al., 2003). Furthermore, by comparing periods of different fetal movement pattern within the fetus, some of the eventual effect of fetal sex was controlled.

### 4.3 Interpretation and relation to other studies

The high intra‐ and inter‐observer reliability indicate that the protocol for selecting time series provides reliable results.

Time domain parameters and especially RMSSD were superior to spectral domain parameters with regard to reliability. This is in accordance with findings in adults (Pinna et al., 2007) and a study by Van Leeuwen et al, who, based on MCG, found RMSSD as more consistent within the same fetus compared to SDNN (Van Leeuwen et al., 2013). However, in that study, fetal movements were not assessed, and absolute (CV) and relative (ICC) reliability were not estimated. Spectral domain parameters are more sensitive to missing beats, extrasystoles, noise and random changes in heart rate than time domain parameters, which is also evident, even in our highly standardized setting. However, the spectral analysis offers the opportunity of identifying the underlying rhythms like HF‐power, which may be related to the activity of the parasympathetic system (Task Force, 1996).

We included all‐time series fulfilling our predefined criteria, as we believed this to be most clinically relevant. Therefore, analyses of unrestricted fetal movements contained nearly 50% of the total recording time, while the analyses of specific movement patterns contained from 15–20% of the total recording time. Despite this advantage in the amount of included time series, the unrestricted movement pattern displayed comparable or even lower reliability results than during specific fetal movements. This supports the hypothesis that standardizing is an important aspect of reliability.

In general, we found ICC’s high, while CV’s were more dependent on a group of analysis (time domain vs. spectral domain) and also the degree of standardization (specific fetal movement patterns vs. all fetal movements). From a physiological perspective, these findings indicate that the regulation of the fetal heart rate is highly developed. The high relative reliability (ICC’s) indicates that the regulation of fetal heart rate is influenced by individual fetal factors and the variance between fetuses is relatively high compared to the variance within each fetus. The fact that CV’s are above 0%, even in this standardized method, indicate that the cardiac regulation is constantly adjusted also within these 20 min recording.

FHRV during FRM generally achieved the highest reliability. However, same‐recording reliability was higher than day‐to‐day reliability, yet, the evaluation of day‐to‐day reliability was based on as low as two‐time series, while the prevailing movement pattern in some recordings deviated from the assessed pattern. By restricting the day‐to‐day analyses to fetuses demonstrating the same prevailing fetal movements pattern at day 1 and day 2, reliability increased in most parameters. This indicates, that FHRV is affected by not only the actual movements but also other factors such as the movements just before and after the included time series. This finding supports that the regulation of FHRV is very complex and that the fetal state properly explains some of the variations from day to day. Nevertheless, these sub‐analyses were based on a few observations.

**FIGURE 4:**
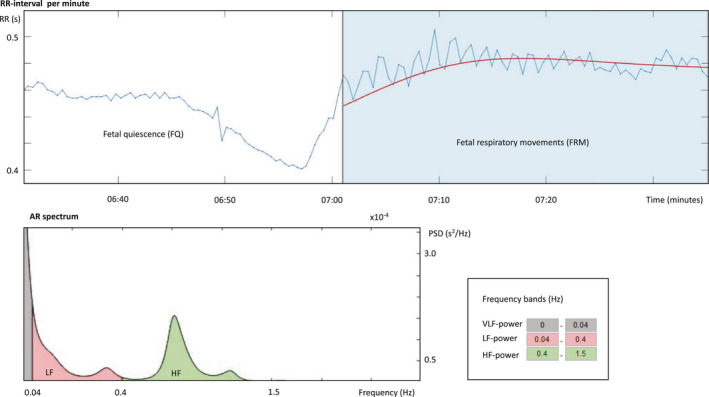
Example of fetal respiratory sinus arrhythmia, where the AR spectrum represents the blue‐coloured part of the upper graph, showing fetal RR‐intervals (seconds) in relation to time (minutes)

Maturation of the autonomic cardiac regulation is supported by our results, as most parameters increased through GA. Furthermore, we found evidence of a fetal respiratory sinus arrhythmia as FRM were associated with increased RMSSD, increased HF‐power, and the occurrence of definite HF‐peaks corresponding to the frequency of FRM, which further underlines the hypothesis of fetal respiratory sinus arrhythmia (Figure 4). In adults, HF‐power and RSA are closely linked to efferent vagal cardiac activity (Katona & Jih, 1975; Katona et al., 1970). In fetuses, this is less studied. However, in the chronically instrumented fetal sheep, it has been shown that vagotomy resulted in a major reduction of RMSSD of approximately 70%, whereas SDNN reduced approximately 30% (Dalton et al., 1983; Lear et al., 2020). Furthermore, sympathectomy resulted in a significant decrease in SDNN, but not RMSSD (Lear et al., 2016). This is in accordance with the hypothesis in adults that of RMSSD primarily reflects vagal activity, and SDNN reflects both sympathetic and vagal activity. Additionally, it has been shown that vagal activity plays a major role in heart rate regulation, especially during hypoxia in fetal sheep. (Giussani, 2016; Giussani et al., 1993).

The association between FHRV parameters of vagal activity and FRM may not solely be caused by shifts in vagal tone in relation to respiratory phases, but may also be caused by other factors such as the mechanical changes in the fetal thoracic pressure due to diaphragmatic movements (Bernardi et al., 1989).

### 4.4 Perspective

Future studies in FHRV need a method that is highly accurate, including caution towards artifact correction, stationarity of mean RR, and fetal movements. Adding fetal movements into the analyses seems to improve reliability and add important information into the interpretation of FHRV. It is advisable to evaluate whether longer recordings and thereby the opportunity of standardizing not only the actual, but also the prevailing movement pattern, improves day‐to‐day reliability.

We detected fetal movements by ultrasound. Other alternatives may include actocardiography, a method revealing the fetal heart vector and thereby gross fetal movements (Brandle et al., 2015), and magnetomyography, which reveals muscle activity by recording magnetic fields and thereby diaphragmatic activity during FRM (Gustafson et al., 2011).

Beat‐to‐beat FHRV in compromised fetuses is poorly investigated; thus, exact guidance in clinical and scientific usage of FHRV must be postponed until further evidence is provided. FHRV in compromised fetuses is a highly significant area of future clinical research.

## 5 CONCLUSION

Analyses of FHRV constitute an investigational tool that has scientific and clinical potential. The increase in RMSSD and HF‐power during FRM indicates that the coupling between respiration and the vagal‐driven RSA is present also in the fetus. This is true despite the lack of respiratory function, and significantly different pulmonary pressure conditions, as compared to neonates and adults.

Standardizing the method is of major importance. Adding fetal movements and especially FRM into the method seems to improve both reliability and, most importantly, the understanding of fetal heart regulation and autonomic function.

## CONFLICT OF INTEREST

No conflicts of interest.

## AUTHOR CONTRIBUTIONS

Zizzo AR, Uldbjerg N, Kirkegaard I, Hansen J, and Mølgaard H contributed to the conception and design of the project. Zizzo AR did the inclusion of participants, obtained recordings, and performed the analyses. Kirkegaard I and From Reese C contributed to the intra‐ and inter‐observer analyses. Zizzo AR did the statistical analyses. All authors contributed to the interpretation of results, writing process and revised the paper critically for important intellectual content. All authors gave final approval of the version to be submitted, agreed to be accountable for all aspects of the work, and are designated as the authors who qualify for authorship.
